# The influence of referent type and familiarity on word-referent mapping

**DOI:** 10.1371/journal.pone.0219552

**Published:** 2019-07-10

**Authors:** Jia Hoong Ong, Alice H. D. Chan

**Affiliations:** Linguistics and Multilingual Studies, School of Humanities, Nanyang Technological University, Singapore, Singapore; University of Nottingham, UNITED KINGDOM

## Abstract

Is our memory for pairs of items dependent on item characteristics? The present study explores this question using a word learning paradigm; specifically, we examined whether referent characteristics, such as referent type (face/object) and familiarity (known/unknown), may influence word-referent mapping. Moreover, we examined this effect across two test sessions to determine if the influence of referent characteristics might be more pronounced over time, and across two age groups (young vs. older adults) to determine if there might be age-related differences. Participants were presented with pseudoword-referent mappings in four referent conditions (face/object × known/unknown) and then were tested with a recognition task immediately after learning, and again after a short delay. Our findings indicated that names for faces were not learned better than names for objects, despite previous literature suggesting that faces are processed differently. We also found that known referents (defined as having a pre-existing label for a referent) were learned better than unknown items but this familiarity advantage was only observed for faces and not for objects. While there were several age-related findings, these might be due to the longer delay between the immediate and delayed tests among the older adults relative to young adults. Taken together, our results suggest that certain referent characteristics do interact and influence our learning of and memory for such pairings.

## Introduction

One aspect of word learning requires learners to map a word to a meaning referent (hereafter word-referent mapping). Previous word-referent mapping studies have focused on the word component, for example, by investigating whether the phonology of the label affects word learning [1]; the learner component, for example, by investigating learners’ assumptions or biases [2]; as well as the mapping component such as by examining whether the learning mechanism is domain-general or domain-specific [3,4]. The present study focuses on the referent component to address whether our learning of and memory for the links between names and referents differ according to referent characteristics. Put differently, we explore whether associative memory may vary for different items.

Studies on second language (L2) vocabulary learning have identified several factors that affect vocabulary learning. One such broad category is interlexical factors such as similarity of word form and meaning relations between first language (L1) and L2 words [5], such that a high degree of similarity of form and meaning between the two languages (e.g., *forest* in English and *forêt* in French) is said to be facilitative. Another broad category that affects L2 vocabulary learning is intralexical factors, such as pronounceability and length of the word form [6]. L2 vocabulary learners are constrained by their L1 phonology, which may explain, for example, why Japanese adults have difficulties differentiating English words such as *rice* and *lice*. Referent characteristics such as abstractness and familiarity of the meaning appear to play a role too [6,7]. We investigated the effect of two referent characteristics on word-referent mapping in the present study—referent type (learning names of faces vs. objects) and familiarity (whether learners have a pre-existing label, i.e., whether the referent is known). Moreover, we explored whether the effect of referent characteristics (if any) might be more evident over time by comparing their performance across two test sessions, and whether there might be age-related differences in such learning with two groups of participants, young and older adults.

Concerning referent type, names for faces, or proper names, are said to be represented differently than common names or other semantic information. In a seminal study, McWeeny and colleagues demonstrated that, when shown a picture of a face, learners had poorer recall the name associated with the face than the occupation, even when the same label was used (e.g., Mr Baker vs. a baker) [8]. Furthermore, there are reports of a double dissociation between common name anomia (i.e., an inability to recall names) and proper name anomia [9]. Proper names are also more prone to the tip-of-the-tongue (ToT) phenomenon—a failure to retrieve the label given a referent—than common names [10–12]. While the evidence suggests that the representation for proper names and common names are different, it is unclear why this is the case. One possibility relates to how the referent is processed; for example, faces are processed more holistically than nonfaces [13,14] and appear to be more salient and attention-grabbing than nonfaces given their biological and social significance [15–17]. Given limited cognitive resources [18], it is plausible that learners may spend more resources encoding a face than an object, which would result in a weaker face-name link and therefore making it more susceptible to retrieval failure. Moreover, proper and common names also differ in their retrieval level. Whereas retrieving the name of a face requires one to retrieve the label at the exemplar level (e.g., *Mr Baker*), retrieving the name of an object enables one to retrieve the label at the category level in which there are multiple possibilities (e.g., *dog*, *mutt*, *hound*, *canine*, etc.), which would facilitate the retrieval process. Indeed, when one is given the opportunity to label faces with more than one name, such as by naming the face either by their real name (e.g., *Harrison Ford*) or a character for which they are known (e.g., *Indiana Jones*), the ToT phenomenon for proper names decreases [19]. Given these differences, it is unclear whether the two may also differ in terms of their learning and retention; it may be the case that due to differences in processing demands, the mapping strength between different referent types and their labels may also differ.

Concerning referent familiarity, there are equivocal findings on whether having a pre-existing label for a referent (i.e., a known referent) facilitates the learning of another label for the same referent [20,21]. Whereas children aged between 3 and 5 were able to learn foreign words for familiar objects, only the older children could do so for unfamiliar objects [20]. However, with additional presentations, the younger children could as well, suggesting that even though it is typically more challenging to learn labels for unfamiliar referents, it is nonetheless possible [20]. On the other hand, some have reported that children acquire foreign labels for familiar and novel objects equally well [21]. Such equivocal findings may be attributed to methodological differences (e.g., number of learning trials, duration of exposure during learning, etc.). It may also be attributed to the fact that such studies do not typically probe whether the referents are truly known/unknown by the learners (e.g., an unknown referent may not actually be unknown to certain learners or learners may conjure up names for the referent) but instead referents are assigned as such *a priori* by the experimenters. It is thus important to address this assumption, for example, by including a stimulus check to ensure the correct assignment of the stimuli to their category according to each participant’s lexical knowledge. According to studies in L2 vocabulary acquisition, a familiarity advantage is seen because learners would have to relabel familiar meanings only whereas they would need to construct a new concept and learn a label for unfamiliar meanings, which is arguably more challenging [6,7,22]. Thus, we argue that a known referent may incur less processing cost, leaving learners with more cognitive resources to process and map the label to the known referent. It remains to be seen, however, whether familiarity and referent type may interact and influence the word learning process. That is, the familiarity advantage may be different for faces than for objects, given that the two referent types are processed and represented differently.

Assuming that referent characteristics (either type or familiarity or an interaction of both) do influence the word-referent mapping process, then two questions are further posited: is the influence (i) stable over time and (ii) more evident for certain learners? Concerning the former, if the association formed between the referent and label (if any) is weak due to certain referent characteristics, then it is likely that the referent-label association would be more prone to be forgotten after a delay. To examine this, the associations between labels and referents were assessed at two time points in the present study: one immediately after learning and again after a short delay.

It may also be the case that those with less cognitive resources such as older adults will be affected by the influence of referent characteristics to a greater degree. Previous studies indicate that older adults have poorer associative memory than young adults, such as in learning names for faces [23–25], but the two show similar recognition of faces and of names separately [26,27]. This led some to propose the associative deficit hypothesis [26,27], which is the proposal that older adults may have difficulty implementing strategies to associate two arbitrary components. Some suggested that older adults tend to hyper-bind [23,25,28], that is, form spontaneous associations between items during learning which may lead to more interference during retrieval. Regardless of the reasons, evidence suggests that older adults consistently show poorer performance than young adults in paired-associate learning. Here, we extend previous research by investigating whether the age-related decline in forming associations between referents and labels may be modulated by referent characteristics such as type and familiarity. If certain referent characteristics impose less processing demands on the word-referent mapping process, then age-related differences may be seen for certain referents.

In summary, the present study explores whether two referent characteristics–referent type (face/object) and familiarity (known/unknown)—influence word-referent mapping, which would address whether the link strength between a referent and its label is partly influenced by referent characteristics. The study also explores whether the influence (if any) is stable across time by comparing learning performance at two time points and across learners with different cognitive resources by comparing the performance of young and older adults.

## Methods

### Participants

The final sample consisted of 50 Singaporean English-Chinese bilinguals, with an equal number of young and older adults. The sample size was determined from previous studies on comparing young vs. older adults on paired-associate learning tasks [23,29]. The young adults (17 females; M_age_ = 22.32, SD = 2.25, Range = 20–28) were students from a local university in Singapore whereas the older adults (14 females; M_age_ = 66.80, SD = 4.17, Range = 60–77) were Singaporean residents recruited from the community. The older adults were screened for cognitive impairment using the Mini-Mental State Examination, Second Edition (MMSE-2) [30] and they scored at least 26 out of 30, indicating that all were cognitively healthy at the time of testing. Participants provided their written informed consent prior to participating and they were reimbursed for their participation. Nanyang Technological University Institutional Review Board approved the study protocol and all methods were performed in accordance with the relevant guidelines and regulations.

### Tasks & stimuli

#### Name learning task

There were 24 word-referent pairings in the name learning task. The words were disyllabic pseudowords, synthesised using Mac OS X Speech Service with the voice *Samantha*. Disyllabic pseudowords were chosen because they allow for greater variation in phoneme order, which would enable us to select pseudowords that do not sound like English or Chinese, the two languages known by the participants (see S1 Table for a list of the pseudowords used). The pseudowords were presented auditorily to reflect how we typically learn names for faces (e.g., in conversations, in films, etc.).

There were two types of referents: faces and objects. Within each referent type, six were known referents (i.e., faces of famous people and everyday objects) and six were unknown referents (i.e., faces of unknown people and novel objects). Thus, there were four referent conditions: known-faces, unknown-faces, known-objects, and unknown-objects. The faces were all of East Asian descent (i.e., identical to the participants) and there was an equal number of male and female faces. The known-faces and unknown-faces were matched in gender and relative age. Four of the known-faces for the older participants were different from those for the younger participants as a pilot study with a different sample revealed age-related differences in their recognition of known-faces. The known-objects were highly familiar items (e.g., pen, chair, pot, etc.) whereas the unknown-objects were novel objects for which participants may not immediately have a label. Familiarity for all the referents was determined for each participant using a questionnaire at the end of the experiment (see below). Images for the referents were either sourced from databases (for the unknown-objects [31] and for the unknown-faces [32]) or from the Internet (for the known-faces and known-objects); see S1 Fig for a representative sample of the images used in each condition. Images of the faces were cropped such that only the face and hair were shown. All the images were edited to have a white background and scaled to 400 × 400 pixels. Two lists of 24 word-referent pairings were formed by randomly pairing the words and the referents. Participants were randomly assigned to a list prior to the name learning task.

The name learning task consisted of three phases: learning, immediate test, and delayed test. At the start of the learning phase, participants were instructed to learn the names of objects and the nicknames of people in an alien language. The 24 word-referent pairings were then presented serially with each pairing presented twice overall. On each learning trial, an image was presented visually on the computer screen for 4.5s and the name for the referent was presented auditorily via headphones 2s after the image was presented. The inter-trial interval was 0.5s. The presentation order of the pairings was pseudorandomised such that no two consecutive pairings were the same. Participants were given an opportunity for a break after every 12 learning trials. After the learning phase, participants completed the immediate test phase, which was a six-alternative forced-choice (6AFC) task. In every test trial, participants were presented with a word auditorily and they had to choose which of the six images on the screen corresponded to the word. The target image was always present along with five distractor images sampled from the same referent condition (e.g., if the target image was from the known-face condition, then all the distractor images were also from the known-face condition). Each pairing was tested once, with the order of the test trials randomised. Prior to the start of the actual task, participants were given two practice trials with pairings not used in the task. A delayed test, of which participants were not informed beforehand, was administered at the end of the experiment, after a series of cognitive tests (see below). The delayed test had the same structure as the immediate test. In general, the duration (in mins) between the immediate and the delayed test was longer among the older adults (M = 44.88, SD = 19.58, Range = 14–99) than the young adults (M = 35.96, SD = 5.91, Range = 28–48), *t*(48) = 2.18, *p* = .034, a point to which we will return in the Results and Discussion sections. After the delayed test, participants completed a post-task questionnaire to probe their familiarity with the referents used in the task (see S1 Appendix for the questions). The questionnaire was completed after the delayed test to ensure (i) that participants did not have the opportunity to conjure up names for stimuli prior to the word learning task; and (ii) minimal exposure to the stimuli, especially the unknown stimuli, prior to learning as previous studies have shown that exposure alone facilitates name learning for novel objects [33]. In the questionnaire, participants were asked whether they recognised each referent, and if so, whether they could name the referent (in English for objects and the real names for the faces). Their response to each referent was coded offline as “Don’t Know”, “Familiar but can’t recall the name”, or “Know” (regardless of whether the participants recalled the name correctly).

#### Measures of general cognitive abilities

To ensure that participants were within the normal range in their general cognitive abilities, we measured participants’ nonverbal intelligence (Test of Nonverbal Intelligence 4^th^ Edition, TONI-4) [34] and English receptive vocabulary (Peabody Picture Vocabulary Test 4^th^ Edition, PPVT-4) [35]. Their scores of each of the test were transformed to age-normed standard scores.

### Procedure

Participants completed the tasks in the following fixed order: (i) name learning task; (ii) immediate test; (iii) TONI-4; (iv) PPVT-4; (v) delayed test; and (vi) post-task questionnaire. The older participants completed the MMSE-2 at the start of the experiment. Participants were free to take a short break between tasks. The young adults took approximately 1 hr to complete the experiment whereas the older adults took approximately 1.5 hrs.

## Results

### General cognitive abilities

Participants’ nonverbal intelligence performance was within ±2 SD from the mean (Young adults: Range = 91–119; Older adults: Range = 94–125), suggesting no extreme outliers. Young adults (M = 104.20, SD = 9.82) and older adults (M = 105.70, SD = 9.59) did not differ significantly on nonverbal intelligence scores (*t*(48) = 0.57, *p* = .573, *d* = 0.16).

Participants’ English receptive vocabulary performance was generally within ±2 SD from the mean, except for one older adult, who scored slightly below 2 SD (Young adults: Range = 91–127; Older adults: Range = 68–113). An independent sample *t*-test revealed that older adults (M = 91.64, SD = 11.53) scored significantly less than the young adults (M = 101.5, SD = 9.18) on English receptive vocabulary (*t*(48) = 3.35, *p* = .002, *d* = 0.95), presumably due to a later acquisition of English by older adults in Singapore. Indeed, from the demographic questionnaire, we found a significant difference between age group and whether they grew up speaking English (*χ*^*2*^(1) = 7.03, *p* = .008): whereas most young adults indicated that they grew up speaking English (21 vs. 4), less than half of the older adults did so (11 vs. 14). Nevertheless, all participants understood the instructions of each task in the present study, which was administered in English.

### Name learning task

Participants’ performance on the name learning task was based only on referents that were rated as “Don’t Know” and “Know” as indicated in the post-task questionnaire. That is, we took into account each participant’s familiarity with the referents to decide which referents were truly known and which were truly unknown (e.g., to avoid cases where participants might conjure a label for what was *a priori* assumed to be an unfamiliar object). Referents rated as “Familiar but can’t recall the name” were discarded from analysis (Overall rate: Young adults = 7.36%; Older adults = 8.33%) since we were only interested in the learning of names of referents for which participants have a label (as in the case of “Know”) or not (as in the case of “Don’t Know”).

Table 1 reports the descriptive statistics of the mean proportion correct for each referent condition by age group and test session. We first examined whether participants performed above chance for each referent condition by performing a series of one-sample *t*-tests against chance performance (1/6). Performance on all the referent conditions was significantly above chance for both young and older adults, which suggests that they successfully learned the names at the group-level.

**Table 1:** Descriptive statistics of the proportion of correct responses for each referent condition by age group and session. *t*-values represent the *t*-statistics from the one-sample *t*-tests against chance performance (1/6).

Referent Condition	Mean (SD)	95% CI	*t*(24)	*d*
Young adults: Immediate test				
Don’t Know–Face	0.55 (0.29)	0.44–0.67	6.64***	1.33
Don’t Know–Object	0.58 (0.33)	0.45–0.71	6.28***	1.26
Know–Face	0.73 (0.30)	0.61–0.85	9.22***	1.84
Know–Object	0.69 (0.22)	0.61–0.78	11.80***	2.36
Young adults: Delayed test				
Don’t Know–Face	0.55 (0.31)	0.43–0.67	6.20***	1.24
Don’t Know–Object	0.54 (0.31)	0.41–0.66	5.89***	1.18
Know–Face	0.78 (0.23)	0.69–0.87	12.52***	2.70
Know–Object	0.67 (0.23)	0.58–0.76	10.85***	2.17
Older adults: Immediate test				
Don’t Know–Face	0.42 (0.29)	0.30–0.53	4.35***	0.87
Don’t Know–Object	0.47 (0.29)	0.36–0.59	5.17***	1.03
Know–Face	0.58 (0.32)	0.46–0.71	6.44***	1.29
Know–Object	0.44 (0.25)	0.34–0.53	5.45***	1.09
Older adults: Delayed test				
Don’t Know–Face	0.32 (0.24)	0.23–0.41	3.19**	0.64
Don’t Know–Object	0.35 (0.32)	0.22–0.47	2.81**	0.56
Know–Face	0.45 (0.32)	0.33–0.58	4.51***	0.90
Know–Object	0.39 (0.26)	0.29–0.49	4.36***	0.87

** *p*< .01,

*** *p*< .001

To examine whether referent type and familiarity influenced name learning performance, the data were fitted using mixed effects logistic regressions using the *lme4* package [36] in R [37]. The dependent variable was Accuracy, a binary categorical variable (Correct/Incorrect). All the predictors—Session (Immediate/Delayed), Age Group (Young/Older), Familiarity (Don’t Know/Know), and Type (Face/Object)—were effect-coded. We first modelled the data with all four fixed effects and all possible interactions between them. We also included scores on PPVT and TONI (both of which were centred) to the model as fixed effects. Model selection was then done using backward elimination, starting with higher order interactions and dropping the terms one at a time if they do not significantly improve model fit. The final model consisted of Age Group, Session, Type, Familiarity, Vocabulary, Age Group × Session, Age Group × Familiarity, and Familiarity × Type (see Table 2). As random effects, we entered random by-subject and by-item intercepts. Pairwise comparisons were conducted using *lsmeans* package [38] in R.

**Table 2:** Output of the mixed effects logistic regression model.

Predictors	Estimated ß	Std. Error	*z*-value	*p*-value
Intercept	0.22	0.15	1.43	.153
Age Group	-0.92	0.28	-3.25	.001
Session	-0.22	0.10	-2.30	.021
Type	-0.10	0.17	-0.59	.558
Familiarity	0.39	0.16	2.49	.013
Vocabulary	0.02	0.01	2.49	.013
Age Group × Session	-0.41	0.19	-2.10	.036
Age Group × Familiarity	-0.47	0.21	-2.25	.024
Familiarity × Type	-0.84	0.30	-2.81	.005

**Fig 1:**
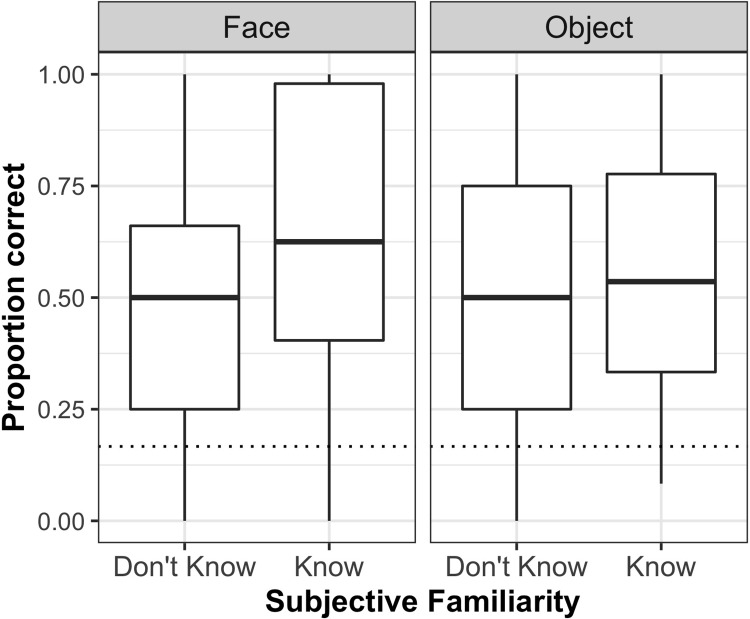
Boxplots illustrating proportion of correct responses by referent type (Face vs. Object) and familiarity (Don’t Know vs. Know) averaged across test sessions and age groups. Dotted line represents chance performance.

We found a main effect of Vocabulary, suggesting that better vocabulary scores led to better overall word learning performance. We also found main effects of Age Group, Session, and Familiarity, all of which were further qualified by two-way interactions: Age Group × Session, Age Group × Familiarity, and Familiarity × Type. Pairwise comparisons indicated that (i) performance was significantly better on the immediate test than the delayed test for older adults (ß_diff_ = 0.43, SE = 0.13, *p* = .002) whereas no significant difference between the two tests was observed among young adults (ß_diff_ = 0.02, SE = 0.14, *p* = .888); and (ii) performance was significantly better on ‘Know’ referents than ‘Don’t Know’ referents (i.e., a familiarity advantage) among young adults (ß_diff_ = -0.62, SE = 0.19, *p*< .001) but not significantly so among older adults (ß_diff_ = -0.16, SE = 0.19, *p* = .405). Importantly, concerning the influence of referent characteristics on word-referent mapping, a familiarity advantage was seen for faces (ß_diff_ = -0.81, SE = 0.22, *p*< .001), and not for objects (ß_diff_ = 0.03, SE = 0.22, *p* = .881) across age groups and test sessions (see Fig 1).

Given that the delay between the immediate test and the delayed test was significantly longer among the older adults than the young adults, any effects related to the Session and Age Group may thus be confounded by the difference in delay duration. We addressed this issue by exploring the immediate test data. Using the same steps of data analysis as described previously, the final immediate test model is shown in Table 3. The model revealed that there was a main effect of Vocabulary such that higher vocabulary scores led to better overall word learning performance, and a main effect of Age Group such that older adults (ß = -0.06, SE = 0.20) had significantly worse overall performance than young adults (ß = 0.65, SE = 0.21). There was also a main effect of Familiarity, which was qualified by a Familiarity × Type interaction. Pairwise comparisons revealed that, similar to the full model, there was a familiarity advantage for faces (ß_diff_ = -0.81, SE = 0.23, *p*< .001) but not for objects (ß_diff_ = -0.15, SE = 0.22, *p* = .502).

**Table 3:** Output of the mixed effects logistic regression model for the immediate test.

Predictors	Estimated ß	Std. Error	*z*-value	*p*-value
Intercept	0.30	0.15	2.04	.041
Age Group	-0.71	0.29	-2.43	.015
Familiarity	0.48	0.16	3.04	.002
Type	-0.14	0.16	-0.88	.376
Vocabulary	.020	0.01	2.46	.014
Familiarity × Type	-0.66	0.32	-2.09	.036

One may argue that the effects that we observed with the referents might be influenced in part by the pseudowords, such as how English- or Chinese-like they sound and how similar they sound to each other. While this is a valid concern, we do not think this is likely since the pseudowords were randomly paired with the referents to create two languages and participants were randomly assigned to one. Nonetheless, we examined this concern directly by asking Singaporean English-Chinese bilingual young adults to rate on a five-point scale how (i) English-sounding, (ii) Chinese-sounding, and (iii) similar sounding to each other the pseudowords were. Ten participants were tested, and their ratings were averaged and included in the analysis. Results showed that the more English-sounding the pseudowords were, the better they were learned, consistent with previous findings [5], and similar-sounding words tend to be learned marginally worse, though these effects were only observed in the overall model and not in the immediate-test model. Importantly, our other findings remained the same and no interactions were observed between the pseudowords and the other predictors (see S2 Table for the output of the models). Thus, we have some assurance that the reported findings in our models were not influenced by the pseudowords themselves.

## Discussion

The present study explores whether our learning of and memory for pairs of items (such as word-referent mappings) may differ according to item characteristics. Two such characteristics (type and familiarity) were examined. We speculated that these characteristics may influence the link strength between the name and the referent, which would affect how well one learns and remembers the pairing. Furthermore, we asked whether the influence of referent characteristics may differ across time and age groups by examining if the influence might be different after a delay between tests and for different age groups, respectively.

Before unpacking the findings, two issues need to be raised, which may influence our interpretation of the results. Firstly, the older adults in our sample have significantly lower English vocabulary proficiency than the young adults, presumably due to a later English acquisition age by the former. This may be an issue, given that vocabulary size does seem to predict word learning performance [39,40]. Indeed, we too found an overall positive relationship between English receptive vocabulary and word learning score. However, the lack of significant interactions involving vocabulary knowledge suggests that the effects reported in our model are not dependent on learners’ vocabulary. The second issue relates to the longer delay between the immediate and delayed tests among the older adults relative to young adults. This was due to the older adults taking a longer time to complete the cognitive tasks and having more breaks between the tasks. While this difference may be a confound in the full model, we could turn to the results from the immediate test to determine if we see a similar pattern of results as the full model.

Learners appear to learn names for faces and objects equally well in general. This is surprising, given that research has shown that faces are processed and retrieved from memory differently than objects [12,13,17]. Our findings suggest that despite all that, the link between names for faces and objects are equally as strong, which implies that when it comes to name learning, faces are not “special” or a different class of entity, as proposed by some in the face perception literature. More generally, it appears that the strength of the relationship between the name and the referent is not influenced by the referent type alone.

We found that names for known items (i.e., having a pre-existing label) were learned better than unknown items, suggesting a familiarity advantage. The advantage arises presumably because a familiar referent is less cognitively taxing to process. Similar proposals were made in second-language (L2) vocabulary acquisition literature, in which it is argued that it would be easier to acquire names for concepts that already exist in the learners’ language [6,7,22]. However, the familiarity advantage was qualified by an interaction with referent type, that is, a familiarity advantage was only seen for faces and not for objects. One possibility for the face-familiarity advantage is due to young and older adults engaging in learning additional names for known faces (e.g., learning character names of actors in films) more often than they do for known objects; thus, the face-familiarity advantage may simply be a consequence of doing a frequent or familiar task. Another possibility is that the known faces (which, in this study, were of celebrities familiar to Singaporeans) may have elicited particular emotions—either positive or negative—leading to better learning [41,42]. Moreover, while effort was taken to ensure that the stimuli of the known-faces and the unknown-faces were matched as closely as possible in the present study, the known-faces had more expressive emotions than the unknown-faces, who mostly had a neutral expression. In other words, the face-familiarity advantage seen in the present study may reflect an emotional advantage either from the identity of the faces or the images themselves. Further work is necessary to explore these possibilities.

The face-familiarity finding has clinical implications for memory rehabilitation for (re)learning face-name pairs [43,44]. Specifically, patients may benefit from having prior exposure or familiarisation to the face-name pairing in order to enhance their learning. Moreover, the findings from the present study could inform us about the development of face-name tests, which are argued to be a useful early marker of cognitive impairments such as Alzheimer’s disease [45,46]. The typical face-name tests use novel face-name pairs, which may be difficult for some, such as those with memory problems. Our findings suggest that face-name tests could incorporate trials in which familiar faces are used so that the test becomes more manageable for some patients and therefore the test would be more sensitive to the underlying cognitive impairment.

In contrast to the face-familiarity advantage, we did not find a familiarity advantage for objects. It thus appears that having a pre-existing label does not seem to facilitate the acquisition of a different label for objects, which, at first glance, may be counterintuitive to the findings from L2 vocabulary acquisition [6,7,22]. Yet this may not be the case; although the participants did not have labels for the unknown objects in the present study, the unknown objects might not necessarily be a foreign concept (e.g., “this is a type of toy” or “this is a toy part”). Therefore, they might not need to conceptualise and label the unknown objects, but simply relabel, as they did with the known objects. In that sense, the presence of labels themselves may not be as important as *conceptual* familiarity in predicting the difficulty learners may face in learning L2 vocabulary.

In the comparison between young and older adults, the older adults performed significantly worse overall than young adults, even on the immediate test. This is unsurprising, given that older adults tend to have poorer associative memory [26,27]. We found that older adults showed a decline in performance between immediate and delayed tests, whereas young adults did not. However, this may be confounded by the fact that there was a longer delay between the two tests for older adults than young adults and so care should be taken in interpreting this interaction. We found that, in the full model, older adults apparently did not benefit from the familiarity advantage seen among the young adults (i.e., the Age Group × Familiarity interaction) though this interaction was not seen in the immediate test model. This suggests that older adults do enjoy the same familiarity advantage as young adults, but the advantage among the older adults may have declined as a result of a longer delay between the tests. Taken together, our results thus suggest that while older adults tend to have worse associative memory than young adults, their ability to learn names are not differentially affected by referent characteristics relative to young adults. Put simply, it appears that referent characteristics affect name learning to the same extent among young and older adults.

Despite conducting a pilot study to ensure that the faces were actually known by the population from which the participants were sampled, some items were still not known by our participants. Furthermore, participants had labels for some of the supposedly unknown faces and objects. While the present study discarded those trials, future research should take this into consideration in order to maximise the number of trials contributing to each condition for each participant while still being feasible. It should be noted, however, that when we ran the analysis on participants that have at least half the number of trials in each condition (young adults, n = 19; older adults, n = 20), the pattern of results is similar to what was obtained in the full sample (see S3 Table for the model output). While the statistical significance of some of the effects is marginal in the reduced sample model, the Familiarity × Type interaction remained statistically significant, suggesting that the interaction is fairly robust. In addition to the stimuli, future research could also vary the task used to probe word-referent knowledge and how referent characteristics may influence it. The present study used a recognition task—specifically, learners have to recognise the meaning of the word, which according to some, is the easiest type of word-referent knowledge to form [47]. It would thus be interesting to examine whether a similar pattern of results would be obtained using a recall task, which is arguably more difficult and is said to assess one’s higher-level word-referent knowledge.

In conclusion, we examined whether our memory for pairs of items, such as word-referent pairings, might be dependent on referent characteristics such as referent type and familiarity, and if so, whether the influence would be stable over time and across age groups. We found that names for faces were not learned better than for objects, but a familiarity advantage was observed only for faces and not for objects. We propose that this face-familiarity advantage may be a consequence of a familiar task or that the known faces may have elicited emotions (due to the identity of the faces or the emotions expressed by the faces), leading to stronger associations formed between the faces and the labels. We speculate that the lack of an object-familiarity advantage may be due to the unknown objects still being conceptually familiar to the learners. While some age-related differences were observed, care should be taken in interpreting those differences as they may be confounded by methodological differences between the age groups. Nonetheless, the present study suggests that certain referent characteristics do interact and influence word-referent mapping, which has implications for memory rehabilitation and assessment as well as L2 vocabulary acquisition.

## Supporting information

S1 Table List of 24 pseudowords used in the experiment. (PDF)

S2 Table Output of the mixed effects logistic regression model for the overall and immediate-test that include pseudoword ratings as predictors. (PDF)

S3 Table Output of the mixed effects logistic regression model on participants that have at least half the number of trials in each condition. Relative to the full sample model, some of the effects are marginal in statistical significance, presumably because of a decrease in statistical power. Note however, that the Familiarity × Type interaction remained significant, suggesting that the effect is fairly robust.
(PDF)

S1 Fig A sample of images used in each referent condition. Note that the unknown faces were drawn from [32] and the unknown objects were drawn from [31]. All other images were retrieved from the Internet.
(PDF)

S1 Appendix Questions on the post-task questionnaire to probe familiarity of each stimulus in the experiment. The questions differ slightly by referent type.
(PDF)
