# Supplementary material for: The influence of referent type and familiarity on word-referent mapping
Source: PLoS One. 2019 Jul 10;14(7):e0219552. doi: 10.1371/journal.pone.0219552 (PMC6619823; doi:10.1371/journal.pone.0219552)
Supplement: S1 Table — (PDF) [file pone.0219552.s001.pdf]

**S1 Table. List of 24 pseudowords used in the experiment**

| | | | |
|---|---|---|---|
| Zoohee | Tajo | Yooka | Xasa |
| Yegen | Pigga | Piho | Javlu |
| Lurae | Sigo | Vuzi | Kibu |
| Hura | Boki | Famdae | Gera |
| Kepfi | Jabo | Davi | Goco |
| Igko | Fumi | Brika | Sozin |
