# Supplementary material for: The influence of referent type and familiarity on word-referent mapping
Source: PLoS One. 2019 Jul 10;14(7):e0219552. doi: 10.1371/journal.pone.0219552 (PMC6619823; doi:10.1371/journal.pone.0219552)
Supplement: S2 Table — (PDF) [file pone.0219552.s002.pdf]

S2 Table. Output of the mixed effects logistic regression model for the overall and immediate-test that include pseudoword ratings as predictors.

Overall

**Predictors**	**Estimated ß**	**Std. Error**	***z*-value**	***p*-value**
Intercept	0.22	0.15	1.44	.149
Age Group	-0.84	0.30	-2.75	.006
Session	-0.22	0.10	-2.30	.022
Type	0.01	0.17	0.07	.948
Familiarity	0.35	0.16	2.26	.024
Vocabulary	0.03	0.01	2.27	.023
English rating	0.37	0.13	2.77	.006
Chinese rating	0.14	0.14	0.99	.322
Similarity rating	-1.45	0.82	-1.77	.076
Age Group × Session	-0.41	0.19	-2.09	.036
Age Group × Familiarity	-0.37	0.21	-1.76	.079
Familiarity × Type	-0.87	0.30	-2.90	.004

Immediate Test

**Predictors**	**Estimated ß**	**Std. Error**	***z*-value**	***p*-value**
Intercept	0.30	0.15	2.07	.038
Age Group	-0.64	0.31	-2.05	.041
Familiarity	0.46	0.15	3.00	.003
Type	-0.06	0.16	-0.39	.696
Vocabulary	0.03	0.01	2.21	.027
English rating	0.20	0.17	1.17	.242
Chinese rating	-0.07	0.18	-0.39	.698
Similarity rating	-0.68	0.99	-0.69	.493
Familiarity × Type	-0.71	0.31	-2.33	.020
