# Supplementary material for: The influence of referent type and familiarity on word-referent mapping
Source: PLoS One. 2019 Jul 10;14(7):e0219552. doi: 10.1371/journal.pone.0219552 (PMC6619823; doi:10.1371/journal.pone.0219552)
Supplement: S3 Table — Relative to the full sample model, some of the effects are marginal in statistical significance, presumably because of a decrease in statistical power. Note however, that the Familiarity × Type interaction remained significant, suggesting that the effect is fairly robust. (PDF) [file pone.0219552.s003.pdf]

S3 Table. Output of the mixed effects logistic regression model on participants that have at least half the number of trials in each condition. Relative to the full sample model, some of the effects are marginal in statistical significance, presumably because of a decrease in statistical power. Note however, that the Familiarity × Type interaction remained significant, suggesting that the effect is fairly robust.

**Predictors**	**Estimated ß**	**Std. Error**	***z*-value**	***p*-value**
Intercept	0.20	0.16	1.23	.219
Age Group	-0.83	0.31	-2.70	.007
Session	-0.21	0.11	-1.93	.054
Type	-0.12	0.17	-0.70	.496
Familiarity	0.31	0.16	1.88	.060
Vocabulary	0.02	0.01	2.62	.009
Age Group × Session	-0.49	0.22	-2.28	.023
Age Group × Familiarity	-0.44	0.23	-1.92	.054
Familiarity × Type	-0.71	0.32	-2.21	.027
