# Supplementary material for: The influence of referent type and familiarity on word-referent mapping
Source: PLoS One. 2019 Jul 10;14(7):e0219552. doi: 10.1371/journal.pone.0219552 (PMC6619823; doi:10.1371/journal.pone.0219552)
Supplement: S1 Fig — Note that the unknown faces were drawn from [32] and the unknown objects were drawn from [31]. All other images were retrieved from the Internet. (PDF) [file pone.0219552.s004.pdf]

S1 Fig. A sample of images used in each referent condition. Note that the unknown faces were drawn from [1] and the unknown objects were drawn from [2]. All other images were retrieved from the Internet.

	Face	Object
**Unknown**	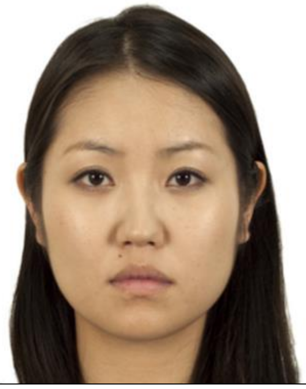	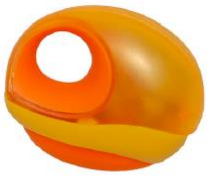
**Known**	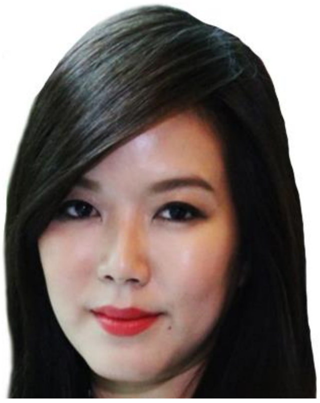	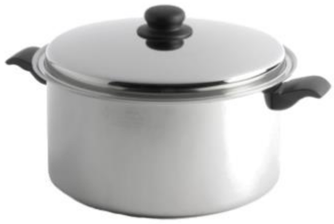

References

1. Ma DS, Correll J, Wittenbrink B. The Chicago face database: A free stimulus set of faces and norming data. Behav Res Methods. 2015;47(4):1122–35.
2. Horst JS, Hout MC. The Novel Object and Unusual Name (NOUN) Database: A collection of novel images for use in experimental research. Behav Res Methods [Internet]. 2016;48:1393–409. Available from: http://dx.doi.org/10.3758/s13428-015-0647-3
