# Supplementary material for: The influence of referent type and familiarity on word-referent mapping
Source: PLoS One. 2019 Jul 10;14(7):e0219552. doi: 10.1371/journal.pone.0219552 (PMC6619823; doi:10.1371/journal.pone.0219552)
Supplement: S1 Appendix — The questions differ slightly by referent type. (PDF) [file pone.0219552.s005.pdf]

S1 Appendix. Questions on the post-task questionnaire to probe familiarity of each stimulus in the experiment. The questions differ slightly by referent type.

Objects:

State whether you're familiar with the following objects and if possible, name them. Please do not search the internet or ask anyone - just try your best to do this without any help!

A. I don’t know what this is

B. It is familiar but I can’t recall its name

C. I know what this is and I can name it

If you answered 'I know what this is and I can name it', please name it here: ____

Faces:

State whether you're familiar with the following faces and if possible, name them. Please do not search the internet or ask anyone - just try your best to do this without any help!

A. I don’t know who they are

B. They are familiar but I can’t remember their name

C. I know who they are and I can name them

If you answered 'I know who they are and I can name them', please name them here: ____
